# Plants as vectors for environmental prion transmission

**DOI:** 10.1016/j.isci.2023.108428

**Published:** 2023-11-10

**Authors:** Christina M. Carlson, Samuel Thomas, Matthew W. Keating, Paulina Soto, Nicole M. Gibbs, Haeyoon Chang, Jamie K. Wiepz, Annabel G. Austin, Jay R. Schneider, Rodrigo Morales, Christopher J. Johnson, Joel A. Pedersen

**Affiliations:** 1 Cellular and Molecular Biology Program, University of Wisconsin – Madison, Madison, WI 53706, USA; 2 U.S. Geological Survey National Wildlife Health Center, Madison, WI 53711, USA; 3 Department of Soil Science, University of Wisconsin – Madison, Madison, WI 53706, USA; 4 Department of Civil and Environmental Engineering, University of Wisconsin – Madison, Madison, WI 53706, USA; 5 Department of Neurology, The University of Texas Health Science Center at Houston, Houston, TX 77030, USA; 6 School of Veterinary Medicine, University of Pennsylvania, Philadelphia, PA 19104, USA; 7 Centro Integrativo de Biologia y Quimica Aplicada (CIBQA), Universidad Bernardo O’Higgins, Santiago, Chile; 8 Department of Environmental Health and Engineering, Johns Hopkins University, Baltimore, MD 21218, USA

**Keywords:** Ecology, Biological sciences, Microbiology, Plant biology, Interaction of plants with organisms

## Abstract

Prions cause fatal neurodegenerative diseases and exhibit remarkable durability, which engenders a wide array of potential exposure scenarios. In chronic wasting disease of deer, elk, moose, and reindeer and in scrapie of sheep and goats, prions are transmitted via environmental routes and the ability of plants to accumulate and subsequently transmit prions has been hypothesized, but not previously demonstrated. Here, we establish the ability of several crop and other plant species to take up prions via their roots and translocate them to above-ground tissues from various growth media including soils. We demonstrate that plants can accumulate prions in above-ground tissues to levels sufficient to transmit disease after oral ingestion by mice. Our results suggest plants may serve as vectors for prion transmission in the environment—a finding with implications for wildlife conservation, agriculture, and public health.

## Highlights

• Abnormal prion protein can enter the roots of plants
• Plants can translocate detectable levels of prions to aerial tissues
• Animals exposed to prion-contaminated plant tissues can acquire disease
• Contaminated plants may represent a route of prion exposure

## Graphical abstract

**Figure:**
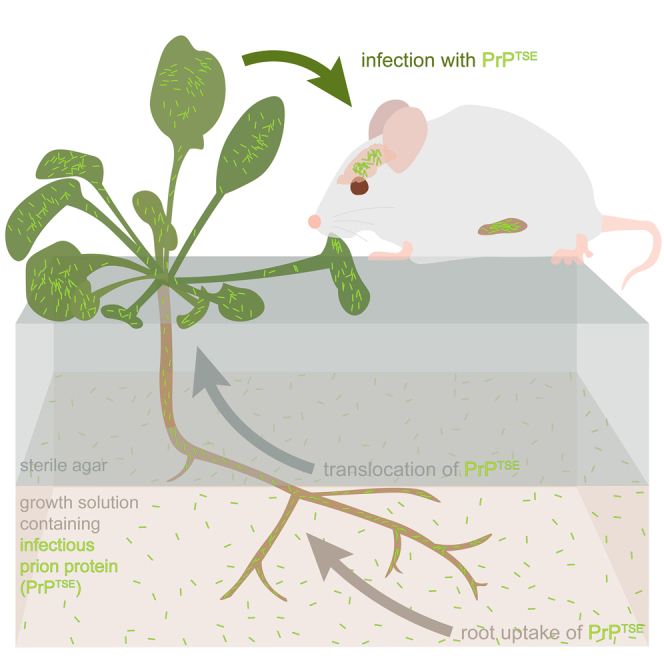

## Introduction

Transmissible spongiform encephalopathies (TSEs, prion diseases) are unusual and invariably fatal neurodegenerative disorders that afflict a variety of mammalian species.^1^ Sheep scrapie was described in Germany and England in agricultural publications in the mid-1700s and may have been known even earlier.^2^ In the 18^th^ and 19^th^ centuries, scrapie outbreaks were often hidden for fear of economic losses,^2^ likely persisting largely uncontrolled across this time. Active management efforts of the last few decades (e.g., surveillance, export, and disposal restrictions), while costly at over $20M/y in the United States, have dramatically reduced scrapie prevalence.^3^ More than 200 years after these initial scrapie reports, bovine spongiform encephalopathy (BSE, “mad cow disease”) emerged in cattle in the United Kingdom and spread to humans, demonstrating the zoonotic potential of ungulate TSEs.^4^ Chronic wasting disease (CWD), a TSE of deer, elk, moose, and reindeer (cervids), was first reported in the U.S. in the late 1960s.^5^ The dramatic spread of CWD across North America, introduction into South Korea,^6^^,^^7^ and recent discovery in Scandinavia^8^ raises considerable public health and food safety concerns. Scrapie and CWD are unique among TSEs in that epizootics can be sustained by direct (animal-to-animal)^9^^,^^10^ and indirect (environmental)^11^^,^^12^ horizontal transmission routes.

**Figure 1:**
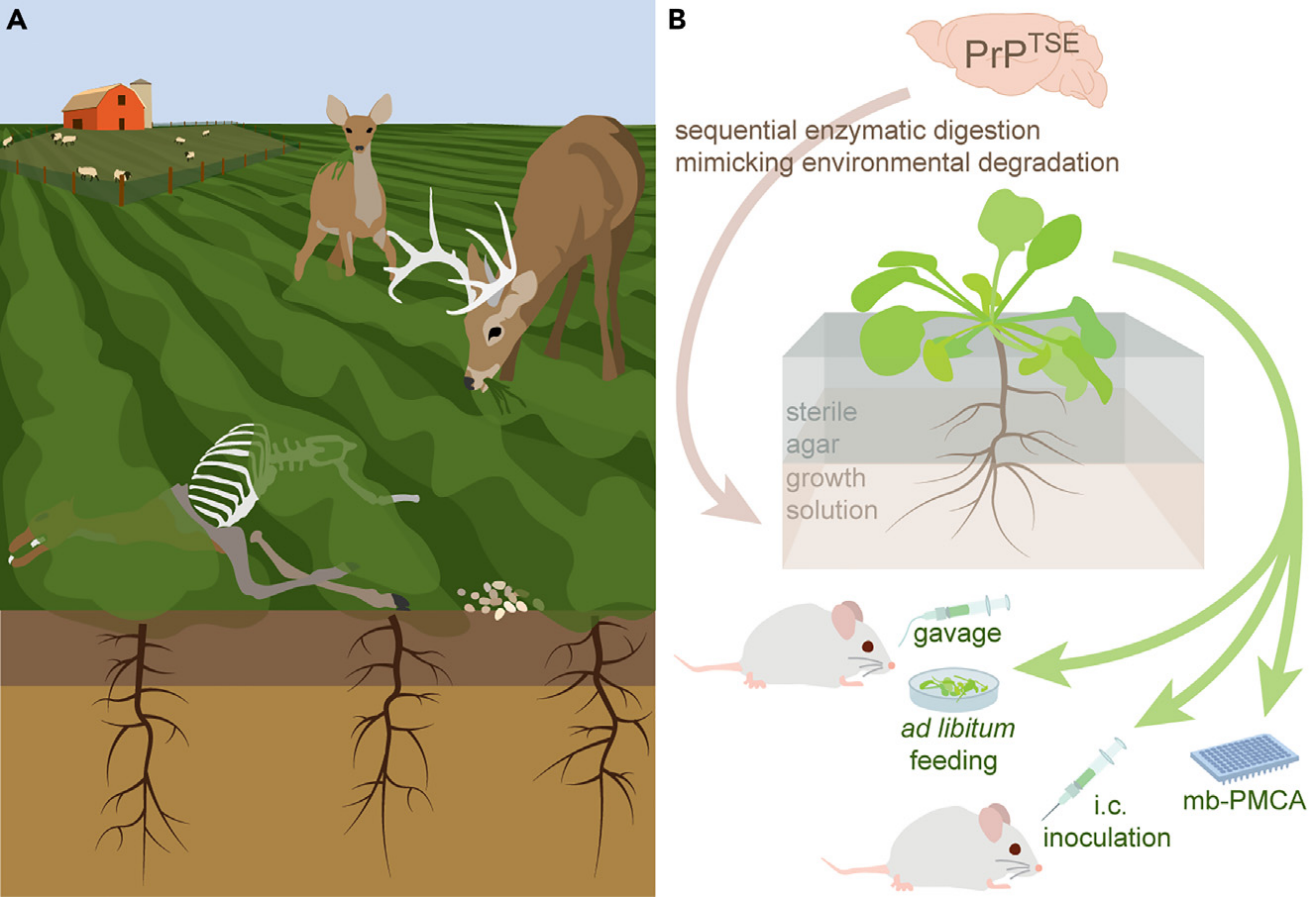
Plants as potential vectors for environmental prion transmission (A) An example of the environmental exposure scenario considered, in which naive, susceptible animals graze on aerial tissues of plants grown in soil contaminated by material containing infectious prions (viz. scrapie for sheep or CWD for deer), including carcass, feces, and other excreta (not shown). (B) Experimental workflow depicting the amendment of plant growth medium with homogenized and digested brain tissue containing pathogenic prion protein (PrP^TSE^). Digestion was performed to simulate environmental decomposition. Stratified growth media were used to avoid contact between aerial plant tissues and the PrP^TSE^-amended medium. Semi-quantitative estimates of the accumulation of PrP^TSE^ in aerial tissues of *Arabidopsis thaliana*, *Medicago sativa* (alfalfa), and *Hordeum vulgare* (barley) were obtained via microplate-based protein misfolding cyclic amplification (mb-PMCA). Infectivity of PrP^TSE^ that had been translocated to aerial tissues was verified via intracerebral (i.c.) inoculation, and the environmentally relevant oral exposure route was tested via gavage and *ad libitum* feeding in CD-1 mice.

Prions, the etiological agents of TSEs, are comprised mostly, if not entirely, of misfolded forms of the prion protein (PrP) that self-propagate via template-driven conversion of the normal, benign cellular conformer (PrP^C^) into the infectious, disease-associated form (PrP^TSE^).^1^ Prions may be released into the environment through urinary, salivary, or fecal shedding from infected animals or decomposition of infected carcasses^13^ (Figure 1). Prions exhibit an extraordinary resistance to inactivation,^14^^,^^15^^,^^16^^,^^17^ and TSE infectivity can persist in the environment for years.^18^^,^^19^^,^^20^ Pathogenic prion protein binds strongly to soil particles, and adsorption to certain soil mineral particles enhances oral transmission of TSEs.^21^^,^^22^ These studies have implicated soil as a natural reservoir for TSE infectivity and led to concern about the accumulation of prions in plants. Prion persistence in, transmission via, and potentiation by components of the environment, coupled with an incomplete understanding of environmental transmission routes, has made eradication of CWD and scrapie a formidable challenge.

The capacity of plants to serve as vectors for prion diseases—to take up, translocate, and subsequently transmit prions—remains poorly understood. Previous efforts have demonstrated root uptake of intact proteins,^23^^,^^24^ but an initial investigation of prion uptake by common wheat (*Triticum aestivum*) did not detect PrP^TSE^ in aerial tissues after short-term exposure of plant roots to prions in contaminated media—potentially due to the high detection limit of the analytical methods employed.^25^ A later report demonstrated some capacity for uptake and translocation of PrP^TSE^ by barley (*Hordeum vulgare*) and showed external contamination of plants with high concentrations of prions (viz. 1% g⋅mL^−1^ infected hamster brain homogenate) can lead to TSE transmission,^26^ but left unanswered whether plants grown in contaminated media could accumulate sufficient amounts of infectious prions in aerial tissues to serve as environmental vectors of prion diseases. Here, we examine the ability of plants to serve as vectors for prion diseases by experimentally investigating the uptake and translocation of PrP^TSE^ from two well-characterized prion strains by several plant species and demonstrate disease transmission via oral exposure to prions accumulated in aerial plant tissues (Figure 1).

## Results

### Sequential degradation of TSE-infected brain material

We assessed prion accumulation in plant tissues using PrP^TSE^ (hyper strain of hamster-adapted transmissible mink encephalopathy) enriched from infected brain matter via a sequential degradation process (viz. digestion with nucleases, lipase, and proteinase K; Figure S1A) designed to simulate decomposition of PrP^TSE^-containing material after entry into the environment. As expected, this method effectively depletes proteins and nucleic acids from brain homogenates, and when TSE-infected tissues are degraded, strong PrP^TSE^ immunoreactivity is retained (Figure S1B). The infectivity of the sequentially degraded brain homogenate is similar to that of proteinase K-treated brain homogenate, with median survival times in a hamster bioassay of 90 and 88 days, respectively, relative to 80 days for untreated infected brain homogenate (Figure S2). This increase in survival time represents an approximately 1-log_10_ reduction in titer after degradation.^27^

### Root uptake of degradation-resistant prion protein

**Figure 2:**
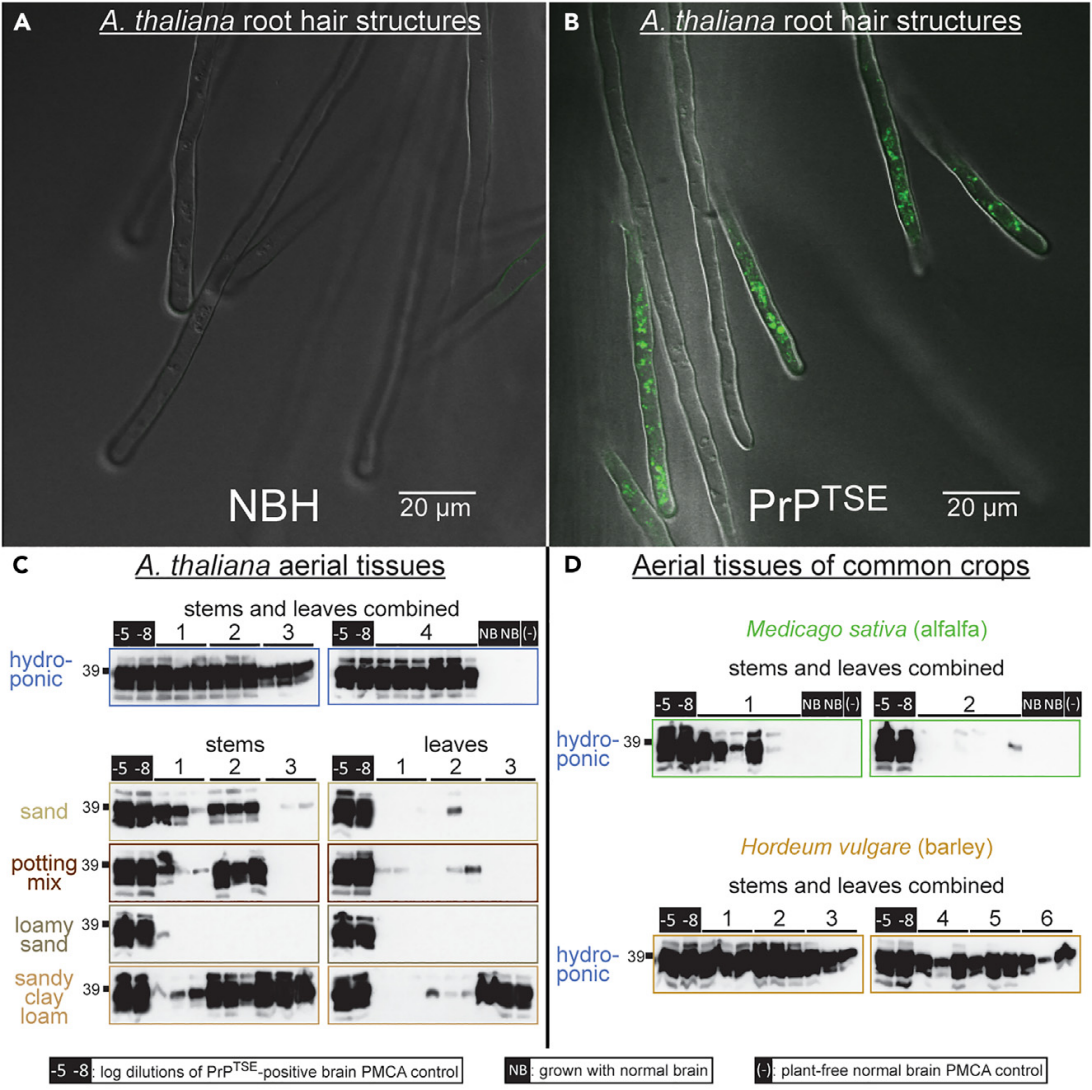
Plants take up pathogenic prion protein (PrP^TSE^) via roots and translocate it to aerial tissues (A–D) Confocal microscopy of *Arabidopsis thaliana* root hair structures after 24-h exposure to media containing (A) normal brain homogenate (NBH) or (B) PrP^TSE^-positive brain homogenate, both of which were experimentally decomposed (via nucleases and lipase) and enzymatically enriched for PrP^TSE^ (via proteinase K) before labeling with Alexa Fluor 488. Aerial tissues of plants grown in PrP^TSE^-contaminated media contain detectable PrP^TSE^ (HY) via serial microplate-based protein misfolding cyclic amplification (5^th^ round shown), in both (C) *Arabidopsis thaliana* grown in various media (hydroponically for 4 days and in sand, potting mix, loamy sand, and sandy clay loam for 22 days); and in (D) the crop species *Medicago sativa* (alfalfa) and *Hordeum vulgare* (barley) grown hydroponically for 25 days. Numbers above lanes indicate individual plants. Brain homogenates were added at 10^−3.0^ to 10^−1.7^ g⋅mL^−1^ liquid media and 10^−1.7^ g⋅mL^−1^ soil. Matrix controls and additional translocation data are shown in Figure S5 and Table S1.

To begin testing whether plants can serve as vectors for environmental prion transmission, we first examined the uptake of PrP^TSE^ into plant roots. We exposed roots of the model plant *Arabidopsis thaliana* to fluorophore-labeled brain homogenates: either normal brain or PrP^TSE^-positive brain. Both were labeled with Alexa Fluor 488 after being subjected to the sequential degradation process described previously. Imaging with confocal microscopy revealed fluorescent green signal within root hairs exposed to the digested PrP^TSE^-positive brain homogenate, but not within root hairs exposed to the digested normal brain homogenate (Figure 2). These results demonstrate that the degradation-resistant PrP^TSE^ can be internalized by the root hairs of *A. thaliana*.

### Detection of degradation-resistant prions in complex plant matrices

Before testing whether plants can subsequently translocate prions to aerial tissues, we established our ability to detect low levels of prions in complex plant homogenate matrices. For this, we employed microplate-based protein misfolding cyclic amplification (mb-PMCA),^22^^,^^28^ a highly sensitive *in vitro* prion conversion and amplification assay, and analyzed a series of 10-fold dilutions (g⋅mL^−1^) of brain homogenate from a clinical end-stage hamster infected with the hyper strain of hamster-passaged transmissible mink encephalopathy prions (titer = 10^9.5^ ID_50_·g^−1^ brain tissue^29^; ID_50_, median infectious dose via the intracerebral route of exposure). We determined the lower limit of detection after five rounds of serial mb-PMCA to correspond to a 10^−15^ (g⋅mL^−1^) dilution of this brain tissue (Figure S3). To determine the effect of complex plant homogenates on detection of PrP^TSE^, we analyzed a series of 10-fold dilutions of PrP^TSE^-positive brain homogenate using serial rounds of bead-assisted PMCA (PMCAb),^30^ a related implementation of PMCA, in the absence or presence of untreated *A. thaliana* stem and leaf tissue homogenate. After five rounds of serial PMCAb, proteinase K-resistant PrP (PrP^res^) was detected through the 10^−13^ dilution in the absence of *A. thaliana* homogenate, compared to 10^−9^ in its presence (Figure S4). The identity of the inhibitor(s) in plant tissue homogenate is unknown, but inhibition of PMCA by components of biological^31^ and environmental^22^ samples has been reported.

### Translocation of degradation-resistant prions

We next determined the extent to which plants can translocate infectious prion protein to aerial tissues after root exposure. We grew the model plant *A. thaliana* on liquid media containing PrP^TSE^ using an adapted version of the high-throughput hydroponic Ice-Cap plant growth method,^32^ preventing cross-contamination of plant aerial stem and leaf tissues by the prion-contaminated growth media. Stem and leaf tissues that had not been in direct contact with prion-containing media were subsequently tested for PrP^TSE^ by serial mb-PMCA. After five rounds of mb-PMCA, some or all *A. thaliana* plants grown in each PrP^TSE^-amended medium held detectable PrP^TSE^ (Figures 2 and S5), but those grown in media containing normal brain homogenate did not (p < 0.001, one-sided Fisher’s exact test based on no false positives in matrix controls, Table S1). To test whether this capacity for prion uptake extends beyond *A. thaliana*, we performed similar experiments using two agriculturally important crop species, commonly consumed by cervids and livestock: alfalfa (*Medicago sativa*) and barley (*Hordeum vulgare*). Following five serial rounds of mb-PMCA, PrP^TSE^ was detected in aerial tissues of both crop species (Figures 2 and S5; p < 0.001, Fisher’s exact test based on no false positives in matrix controls; Table S1). These data suggest that diverse plant species, including both non-mycorrhizal *A. thaliana* and mycorrhizal crop species, can take up, translocate, and accumulate PrP^TSE^ in aerial tissues when grown hydroponically.

To assess plant uptake of prions from contaminated soil, we grew *A. thaliana* in three soils with contrasting properties (loamy sand, sandy clay loam, and sand; Figure S6) and in commercial potting mix, amended with PrP^TSE^-positive brain homogenate. We again grew plants under conditions that prevented cross-contamination of plant aerial tissues from the prion-contaminated media. Aerial tissues of *A. thaliana* tested positive for PrP^TSE^ by mb-PMCA (five rounds) after growth in contaminated sand, sandy clay loam, and potting mix, but not loamy sand (Figure 2). Stems generally appeared to contain more PrP^TSE^ than leaves. No false positives were observed for *A. thaliana* plants grown in these soils amended with normal brain homogenate (Figure S5). These results confirm the findings of an initial report describing PrP^TSE^ uptake from a soil of unreported composition,^26^ and raise new questions about the impact of soil texture on plant uptake. For example, clays have a higher adsorption capacity for PrP^TSE^ (by mass) than does sand,^33^ yet here plant uptake from sandy clay loam appears higher than from loamy sand. Regardless, it appears *A. thaliana* can take up PrP^TSE^ from varied soils and translocate it to aerial tissues.

### Mice exposed to plants grown in media containing degradation-resistant prions

**Figure 3:**
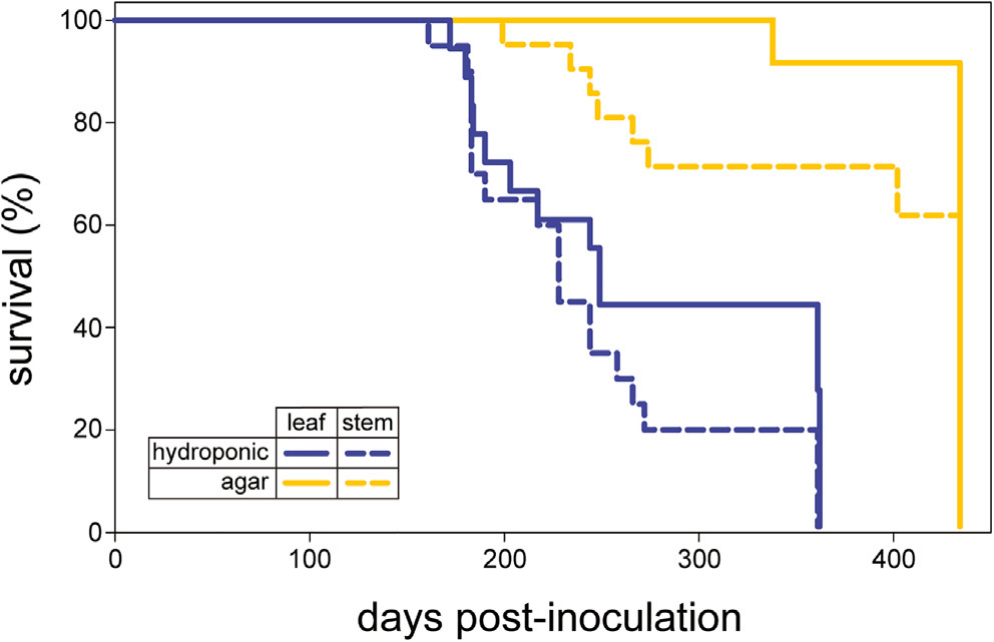
*Arabidopsis thaliana* takes up and accumulates sufficient quantities of PrP^TSE^ in aerial tissues to transmit disease via intracerebral inoculation Survival curves of mice treated with *A. thaliana* aerial tissues (leaves and stems), grown in PrP^TSE^-containing agar (n = 12 and n = 21, respectively) and hydroponic medium (n = 18 and n = 20, respectively). Mice were euthanized on the first day of appreciable clinical disease. Mice inoculated with plant tissue not exposed to prions did not exhibit disease signs.

To determine whether plants can accumulate sufficient PrP^TSE^ to transmit disease, we first intracerebrally exposed CD-1 mice to aerial tissues of *A. thaliana* grown in agar or aqueous substrate containing PrP^TSE^ (RML strain of mouse-adapted scrapie), using the same methods to prevent external contamination of the aerial tissues. Mice were euthanized on the first day of appreciable clinical disease (e.g., kyphosis, lethargy, hindlimb clasp, poor balance). Clinical prion infection was apparent in mice treated with the aerial tissues of *A. thaliana* grown on either hydroponic solution or agar amended with PrP^TSE^ (Figure 3). Disease onset was more rapid and infection rates were higher for mice inoculated with tissues from plants grown hydroponically than those grown on agar, and stems led to faster disease onset than leaves. These data demonstrate plant uptake, translocation, and accumulation of a second prion strain (here: mouse-passaged sheep scrapie prions^34^; previously: hamster-passaged transmissible mink encephalopathy prions^29^), indicating these processes can be expected to occur regardless of prion strain (e.g., scrapie, CWD, and BSE). Overall, these results demonstrate that plants can accumulate sufficient levels of prions from contaminated media to transmit disease by intracerebral inoculation.

**Figure 4:**
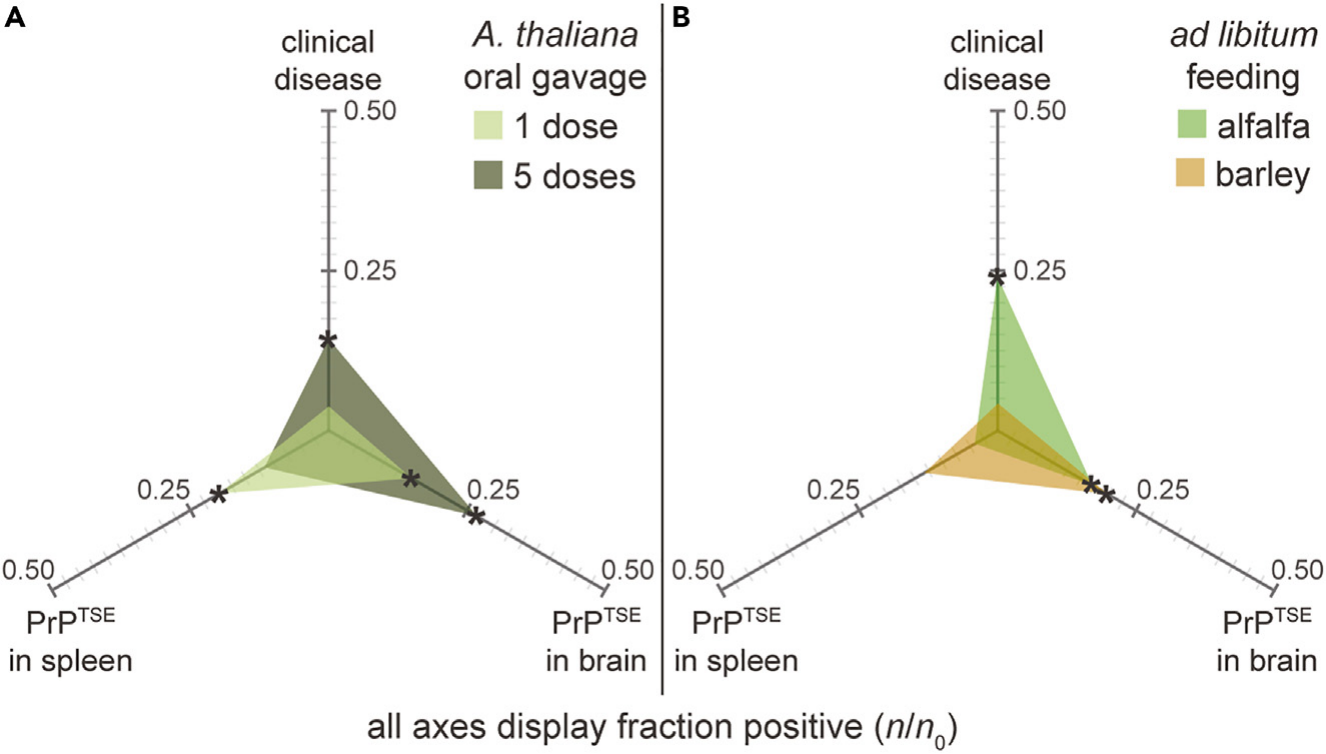
Pathogenic prion protein (PrP^TSE^) accumulates in aerial plant tissues to levels that allow disease transmission via the oral route of exposure Fraction of mice positive for clinical disease and detectable PrP^TSE^ in spleen and brain tissues (via mb-PMCA assay; 3 rounds) after feeding with aerial plant tissues grown in hydroponic media containing decomposed PrP^TSE^-positive brain homogenate. (A) PrP^TSE^ accumulated in aerial tissues of *Arabidopsis thaliana* infects mice after oral gavage (n = 25 to 28). (B) PrP^TSE^ accumulated in alfalfa and barley infects mice after *ad libitum* feeding (n = 23 to 25). **∗** Denotes p < 0.05; one-sided Fisher’s exact test comparing clinical disease or PrP^TSE^ detection in mice fed plants grown in media containing PrP^TSE^-positive brain with control mice fed plants grown in normal brain homogenate (0/30 positive for clinical disease or PrP^TSE^ detection). The prion strain was RML. See Table 1 for additional details.

Finally, to determine whether plants can serve as vectors for environmental transmission of prion diseases, we fed aerial tissues of plants grown in contaminated media to CD-1 mice via oral gavage or *ad libitum* feeding. Here again, we grew *A. thaliana*, alfalfa, and barley on hydroponic media containing PrP^TSE^-infected brain or normal mouse brain homogenate in a manner that prevented external contamination of the aerial tissues. For oral gavage, mice received *A. thaliana* as either a single dose of plant homogenate (equivalent to ∼0.5 g raw plant tissue, based on ∼80% leaf water content by mass^35^) or the same dose per day for 5 consecutive days. For *ad libitum* feeding, mice were provided with raw aerial tissues from the crop species alfalfa or barley at 0.14 g⋅d^−1^ for 30 days. We monitored mice for signs of clinical disease and analyzed brain and spleen tissues for the presence of PrP^TSE^ by mb-PMCA. Strikingly, all feeding treatments resulted in transmission of prion disease, and clinical signs were manifested by a significant proportion of animals in half of the treatment groups (Figure 4; p value <0.05, Fisher’s exact test based on no false positives in control mice fed plants grown in normal brain homogenate; Table 1). Infection rates varied among treatment groups. None of the control mice fed plants grown in normal brain homogenate were positive for clinical disease or PrP^TSE^ in brain or spleen tissue (n = 30). In the oral gavage experiments, the five-dose regimen generated clinical disease at a significant rate (p = 0.048), and the single-dose regimen led to subclinical disease (as evidenced by PrP^TSE^ accumulation in brain and spleen tissues) in a significant proportion of mice (p < 0.05). *Ad libitum* feeding, a more environmentally relevant exposure regime, led to a significant rate of clinical disease in mice consuming alfalfa (p = 0.0061), as well as detection of PrP^TSE^ in brain (p = 0.036). *Ad libitum* feeding on barley did not lead to a significant rate of clinical disease, but a significant proportion of the mice contracted subclinical prion infection (p = 0.017). Interestingly, these plants serve as prion vectors despite the apparent inhibitory effect of untreated plant material on the infectivity of orally administered PrP^TSE^-positive brain homogenate (Figure S7). The factors underlying the inhibitory effect of plants on both oral infectivity *in vivo* (Figure S7) and detection via PMCA (Figure S4) are unclear, but nonetheless the degree of inhibition was not sufficient to prevent transmission.

**Table 1:** Infection rates for mice orally challenged with aerial tissues of *A. thaliana*, alfalfa, or barley grown in media containing sequentially degraded PrP^TSE^-infected or normal brain tissue

Brain material	Plant species	Doses	Route	Clinical disease *n/n*_0_ (p value)^1^	PrP^TSE^ in brain *n/n*_0_ (p value)^1^	PrP^TSE^ in spleen *n/n*_0_ (p value)^1^
PrP^TSE^-infected	*A. thaliana*	1	oral gavage	1/27 (0.47)	**4/27 (0.048)**	**5/25 (0.028)**
	*A. thaliana*	5	oral gavage	**4/28 (0.048)**	**7/26 (0.0032)**	3/25 (0.12)
	alfalfa	30	free-fed	**6/25 (0.0061)**	**4/24 (0.036)**	1/24 (0.50)
	barley	30	free-fed	1/24 (0.44)	**5/25 (0.017)**	3/23 (0.11)
Normal	pooled	–2	–2	0/30	0/29	0/24

1 One-sided Fisher’s exact test comparing clinical disease or PrP^TSE^ detection in mice exposed to prions via diet with control mice fed plants grown in normal brain homogenate; **bolded** when p < 0.05.

2 Pooled control groups; 6 to 8 replicates for brain and 4 to 8 for spleen.

## Discussion

We examined whether plants could serve as vectors for prion diseases by experimentally investigating the complete process: root uptake, translocation, and subsequently transmission of prions via the environmentally relevant oral route. Others have provided evidence for some of these steps (viz. uptake via roots and translocation of PrP^TSE^ by barley and oral transmission after external contamination of plants with high concentrations of prions^26^), but the question of whether plants grown in contaminated media could accumulate sufficient amounts of prions in aerial tissues to serve as environmental vectors of prion diseases remained unanswered. Here, we have shown that plants can take up, translocate, accumulate, and deliver enough prions to infect mice via the oral route of exposure.

It is important to note the present study was conducted using carefully controlled laboratory models of environmental prion uptake and transmission and future efforts to study such processes at field scale in larger animals are certainly warranted. Aspects of these experiments that do not faithfully recapitulate the diseases they model include animal digestive tract structure (viz. mouse versus ruminant), prion strain (viz. lab animal-adapted strains versus authentic stains), and the means by which infectious prions were introduced to plants (e.g., extent of infected material degradation). Nonetheless, we expect the present models do offer key insights into likely behavior in the environment. For example, while the minimum infectious dose of prions in plants should certainly be examined under environmentally relevant conditions, a vanishingly small amount—as little as 300 ng of CWD-infected brain material—is sufficient to infect white-tailed deer when delivered orally.^36^ Additionally, the duration of prion exposure, to both plants and subsequently mice, was transient and compressed here relative to scenarios relevant to the environment or to animal husbandry. Wild plants and crops could accumulate prions over longer periods of time from contaminated soils and serve as vectors for exposure over the lifetime of deer and other animals. Environmental exposures would involve repeated, intermittent intake, which is likely to increase disease incidence relative to a single ingestion event.^37^

At present, our ability to characterize the risk of plants as vectors for prion transmission is impeded by the lack of quantitative information on prion uptake, distribution, metabolism, and excretion in both plants and animals. Nonetheless, our finding of accumulation of two prion strains by a variety of plants grown hydroponically, in agar, or on soil supports the potential for plants to acquire CWD, scrapie, or other prions from the environment and transmit prion disease to susceptible hosts, making plants a plausible vector for prion diseases in wildlife, livestock, and humans. The potential for plants to serve as vectors for prion disease has implications for the disposal of infected carcasses, grazing practices, and the use and transport of potentially contaminated crop materials.

### Limitations of the study

As discussed previously, the present study was conducted using carefully controlled laboratory models of environmental prion uptake and transmission and future efforts to study such processes at field scale in larger animals are certainly warranted. Aspects of these experiments that do not faithfully recapitulate the diseases they model include animal digestive tract structure (viz. mouse versus ruminant), prion strain (viz. lab animal-adapted strains versus authentic stains), and the means by which infectious prions were introduced to plants (e.g., extent of infected material degradation).

## STAR★Methods

### Key resources table

**Table:** 

REAGENT or RESOURCE	SOURCE	IDENTIFIER
**Antibodies**		

3F4 anti-PrP antibody (for hamster)	Millipore	RRID: AB_94253
SAF-83 anti-PrP antibody (for mouse)	Cayman Chemical	RRID: AB_327967

**Biological samples**		

Rocky Mountain Laboratories strain of mouse-passaged sheep scrapie	National Wildlife Health Center	RML
Hyper strain of hamster-passaged transmissible mink encephalopathy	National Wildlife Health Center	HY
Uninfected control brain material (hamster and mouse)	National Wildlife Health Center	N/A

**Chemicals, peptides, and recombinant proteins**		

DNAse I	Worthington Biochemical Corp.	#LS002139, lot S0N12281A
RNAse A	AppliChem	#A2760, lot 8M003055
AY30 lipase	Acros Organics	#2947, lot A0269035
proteinase K-agarose	Sigma	#P9290, lot 037K5159
Freon 113 (1,1,2-trichloro-1,2,2-trifluoroethane)	ChemNet	#CN1099.9
Alexa Fluor® 488 carboxylic acid, succinimidyl ester fluorophore	Life Technologies	N/A

**Experimental models: Organisms/strains**		

Syrian hamsters (*Mesocricetus auratus*; male)	Harlan Laboratories	N/A
CD-1 Swiss mice (*Mus musculus*; male)	Harlan Laboratories	N/A
*Arabidopsis thaliana* ecotype Columbia	Masson Laboratory; UW-Madison	N/A
Barley (*Hordeum vulgare*)	Fedco Seeds	N/A
Alfalfa (*Medicago sativa*)	Fedco Seeds	N/A

**Software and algorithms**		

GraphPad Prism	GraphPad	N/A

**Other**		

Slide-A-Lyzer G2 dialysis cassettes (10 kDa MWCO)	Thermo Scientific	#66380
Potting soil (Miracle-Gro® Organic Choice Potting Mix)	Miracle-Gro	N/A
Loamy sand (Plainfield A)	Dr. Matilde Urrutia, UW-Madison	N/A
Sandy clay loam (Bluestem sandy clay loam)	Dr. Craig Benson, University of Virginia	N/A
Sand (Ottawa sand)	Fisher	catalog no. S23-3
Misonix S-4000 microplate horn (for PMCA assay)	Misonix	N/A

### Resource availability

#### Lead contact

Further information and requests for resources and reagents should be directed to and will be fulfilled by the lead contact, Christopher J. Johnson (cjjohnson@usgs.gov).

#### Materials availability

This study did not generate new unique reagents. Any use of trade, firm, or product names is for descriptive purposes only and does not imply endorsement by the U.S. Government.

#### Data and code availability

• All data reported in this paper will be shared by the lead contact upon request.
• This paper does not report original code.
• Any additional information required to reanalyze the data reported in this paper is available from the lead contact upon request.

### Experimental model and study participant details

#### Animals

Experiments involving animals were conducted at the National Wildlife Health Center of the U.S. Geological Survey in accordance with approved institutional animal care and use committee protocols EP131111R1, EP120822, EP111212, EP080716, and EP130815. Syrian hamsters (*Mesocricetus auratus*; male, weanling age) and CD-1 Swiss mice (*Mus musculus*; male, 4 to 6 weeks of age) were purchased from Harlan Laboratories.

#### Source of prion protein and brains

Disease-associated prion protein (PrP^TSE^) was generated from hamster by intracerebral infection of Syrian golden hamsters with the hyper (HY) strain of hamster-passaged transmissible mink encephalopathy.^29^ Mouse PrP^TSE^ was generated by intracerebral infection of CD-1 Swiss mice with the Rocky Mountain Laboratories (RML) strain of mouse-passaged sheep scrapie.^34^ Brains were harvested from animals displaying clinical signs of disease consistent with TSE and stored at –80°C until used. Non-infected brains were from animals never experimentally challenged or exposed to areas where prion-challenged animals were housed.

#### Sequential enzymatic degradation of brain tissue to simulate decomposition in the environment and enrich the disease-associated prion protein

Brain tissue was processed according to a prion enrichment method we developed for this study that more closely mimics environmental degradation of prion-infected tissues than previously reported protocols.^38^^,^^39^ One gram of prion-infected or non-infected brain tissue (approximately 3 brains) was homogenized in either deionized H_2_O, 1× PBS, or 10 mM HEPES, pH 7.0 at 10^-1.0^ to 10^-0.7^ g·mL^-1^. Brain homogenates (BHs) were then subjected to a series of enzyme treatments aimed at degrading the major classes of biomolecules found in mammalian tissue and enriching proteinase K-resistant prion protein (PrP^res^). All enzyme stocks were prepared and stored per manufacturers’ instructions. First, 10 μg·mL^-1^ each of DNAse I (Worthington Biochemical Corp.) and RNAse A (AppliChem) were simultaneously added to HY-infected or non-infected (controls) BHs and incubated for 24 h at room temperature on an end-over-end mixer. Following nuclease treatment, 100 μg·mL^-1^ of AY30 lipase (Acros Organics) was added to BHs and incubated for 24 h in an identical fashion. Finally, 50 μg·mL^-1^ equivalent of proteinase K-agarose (PK; Sigma) was added to BHs and incubated for 2 to 24 h on an end-over-end mixer at room temperature. Proteinase K-agarose pellets were subsequently removed from samples by low-speed centrifugation (≤ 0.2 *g*), and BH preparations were transferred to sterile microcentrifuge tubes.

Immediately following enzymatic digestion, lipids were extracted from BHs using three parts Freon 113 (1,1,2-trichloro-1,2,2-trifluoroethane; ChemNet) to two parts BH and vigorous shaking on a tabletop shaker (30 min, room temperature). The aqueous upper phase containing PrP^res^ was separated from the denser Freon 113 phase by pipette transfer to a sterile microcentrifuge tube. Brain homogenates were then dialyzed using Slide-A-Lyzer G2 dialysis cassettes (10 kDa MWCO; Thermo Scientific) against three changes per day of ultra-pure, deionized water (pH 7.0) over 5 days at 4°C to remove small molecular mass contaminants and products of enzymatic digestion. Dialyzed BH samples were diluted to a 10^-1.4^ g·mL^-1^ working concentration for all subsequent analyses, unless otherwise stated, and stored at –80°C until needed. All experiments in this study use PrP^res^ prepared by this method, unless specifically stated otherwise.

#### Characterization of sequentially degraded brain tissue

We designed the subtractive enrichment method for PrP^TSE^ based on the physical, chemical, and biological degradative processes to which PrP^TSE^-infected tissues or excretions are generally subjected in the environment (*vide supra*). We compared this new method with a traditional method of prion purification, initially described by Raymond and Chabry.^39^ One HY-infected hamster brain was used to characterize the sequential degradation process.

##### SDS-PAGE and immunoblotting

Lithium dodecyl sulfate and a reducing agent (Invitrogen) were added to BHs to final concentrations of 1× before heating at 95°C for 5 minutes. Thirty microliters of each 4% (g·mL^-1^) BH were run on a pre-cast 12% NuPAGE Bis-Tris gels (Invitrogen) and transferred to a polyvinyl difluoride (PVDF) membrane, following manufacturer’s protocol. PVDF membranes were blocked for 1 h in 5% (g·mL^-1^) powdered milk in Tris-buffered saline with Tween-20 and immunoblotted with monoclonal antibody 3F4 (Millipore) and goat anti-mouse IgG-horseradish peroxidase (Santa Cruz Biotechnology), as previously described.^40^ The reference ladder was Life Technologies SeeBlue plus 2. Imaging was performed using a UVP EC3 imaging system equipped with a Chemi HR 410 camera and VisionWorks LS image acquisition and analysis software (UVP Bioimaging Systems).

##### Protein quantification

Total proteins electrophoresed on 12% SDS-PAGE gels were visualized by Coomassie staining. Gels were placed in a solution containing 0.1% Coomassie stain (Sigma-Aldrich) in 50% methanol and 5% acetic acid (*v/v*) at room temperature for 2 h with rocking and subsequently washed in three changes of 20% methanol, 10% acetic acid (*v/v*) over a 24-h period. Total protein concentrations were determined using the bicinchonic acid assay (Thermo Scientific), following manufacturer’s protocol.

##### Nucleic acid analysis

BH samples (4 μL each) were loaded into a 1% agarose gel cast in 1× Tris acetate EDTA (TAE) buffer containing Sybr Green nucleic acid stain (1:25,000 dilution; Lonza) and electrophoresed for 1 h at 70 V in running buffer (1× TAE buffer with 1:250,000 Sybr Green) using a horizontal gel electrophoresis system (Life Technologies). Nucleic acids were visualized and imaged by UV irradiation (Syngene) using GeneSnap software (Synoptics).

##### Hamster infectivity bioassay

Untreated control, PK-digested, and sequentially degraded BH preparations were diluted to 0.1% (g·mL^-1^) solutions in phosphate buffered saline (pH 7.4). Fifty microliters were intracerebrally inoculated into male, weanling Syrian hamsters (Harlan). Hamsters were monitored daily for the onset of clinical signs and were euthanized when they exhibited significant clinical impairment and neurological signs of disease consistent with a TSE. Survival curves were compared using the Mantel–Cox log rank test (Prism).

#### Plant growth and culture conditions

*Arabidopsis thaliana* ecotype Columbia [Col-0] was used in this study (kind gift from the laboratory of Dr. Patrick Masson, University of Wisconsin – Madison). To meet the specific requirements of individual experiments, various plant growth and culture methods were employed, as described below.

##### Vertical plate agar culture

Plants were grown by this method for use in the root uptake by confocal microscopy experiment. Surface-sterilized seeds were grown on square (100 mm × 15 mm), gridded petri dishes containing 45 mL of nitrogen-free 0.5× Murashige and Skoog (MS)^41^ or 0.5× Linsmaier and Skoog (LS)^42^ basal salt solution (Caisson Labs) supplemented with 15 g·L^-1^ sucrose, 0.4% phytoagar (MIDSCI), and 2.5 mM MES buffer, pH 5.7 (Thermo Fisher). Plated seeds were incubated at 4°C for 4 days to synchronize germination, then transferred to a growth rack (room temperature, 16 h/8 h day/night, 150 μmol·m^-2^·s^-1^ light intensity) for growth in a vertical position.

##### Horizontal plate agar culture

Plants were grown by this method for use in the root uptake experiment and the intracerebral inoculation experiment. Plate and seed preparation and growth conditions for horizontal agar culture were identical to those for vertical plate agar culture (*vide supra*), with the exception that plants were grown in a horizontal position.

##### Six-well plate liquid culture

Plants were grown by this method for use in the intracerebral inoculation experiment. *Arabidopsis thaliana* plants were initially germinated and grown on sterile MS agar via either the vertical or horizontal plate methods (*vide supra*). At four weeks growth, plants were transferred to a hydroponic culture system in which roots were exposed to plant buffer solution (20 mM KCl, 2 mM CaCl_2_, 2 mM MgCl_2_, 10 mM MES, pH 5.7) contained within the wells of a 6-well plate (5 mL of buffer solution per well) and aerial tissues were suspended above each of the wells and out of direct contact with the buffer solution below by a Parafilm barrier (Bemis). Either RML-infected or non-infected mouse BH resulting from the prion enrichment process described above was added to plant buffer solution at a final concentration of 10^-2.0^ to 10^-1.3^ g·mL^-1^ BH. Plants grown by this method were maintained in a humidified chamber constructed by placing the 6-well plate containing plants and media on top of an empty pipette tip box in a deep glass dish filled with 2.5 to 5 cm tap water and enclosing the entire system in plastic wrap for the duration of plant growth. Cultures were aerated daily by gentle manual shaking.

##### Ice-Cap liquid culture

Plants were grown by this method for use in the plant matrix effects on PMCA, PrP^TSE^ translocation, and oral bioassay experiments. The Ice-Cap high-throughput plant growth and tissue sampling system was used as previously described.^32^^,^^43^^,^^44^ Briefly, surface-sterilized plant seeds were sown onto the surface of sterile agar plugs (agar composition identical to that used in vertical plate agar culture above; 200 to 400 μL agar per well, depending on plant species) in 96-well unfritted deep well plates (Fisher). Once seeded, plates were covered with a clear plastic lid (*viz*. lids of flat-bottom 96-well polystyrene plates; Falcon), wrapped in foil, and held at 4°C for 4 days to synchronize germination. Following 4°C incubation, the foil was removed, and seedling plates were transferred to a growth rack (room temperature, constant fluorescent light, 150 μmol·m^-2^·s^-1^ light intensity) for 3 to 5 days, or until seedlings germinated. Once germination occurred, seedling plates were transferred to the Ice-Cap continuous watering system developed by and maintained in the laboratory of Dr. Patrick Krysan at the University of Wisconsin – Madison,^32^ or an adapted version of Ice-Cap using a reservoir watering system maintained at the USGS NWHC. Unlike the classical Ice-Cap approach,^32^ seedling plates were floated in the water-filled upper metal pan reservoir without lower root plates, and plants were allowed to grow at room temperature under constant light until root tissue was observed emerging from the holes in the bottom of each well of the 96-well seedling plates. Seedling plates were then removed from the continuous watering system, and each placed onto a tight-fitting, inverted pipette tip box lid that contained 30-50 mL of 10^-3.0^ to 10^-1.7^ g·mL^-1^ prion-infected or non-infected BH in plant buffer solution, such that only emerging plant roots on the bottom of seedling plates came into direct contact with BH-containing liquid media in the reservoir. Media-containing reservoirs were adhered to seedling plates with tape, plants were grown on a growth rack (room temperature, 16 h/8 h day/night, 150 μmol·m^-2^·s^-1^ light intensity), and cultures were aerated daily by gentle manual shaking.

##### Soil culture

Plants were grown by this method for use in the plant matrix effects on PMCA and PrP^TSE^ translocation experiments. HY-infected or non-infected hamster BH resulting from the prion enrichment method (*vide supra*) was dialyzed against a 1:1 mixture of tap and distilled water and added to a final concentration of 10^-1.7^ g·mL^-1^ to 15 mL of each of the following soils in glass vials: potting soil (Miracle-Gro® Organic Choice Potting Mix), loamy sand (Plainfield A; kind gift of Dr. Matilde Urrutia, University of Wisconsin - Madison), sandy clay loam (Bluestem sandy clay loam; kind gift of Dr. Craig Benson, University of Virginia), and sand (Ottawa sand; Fisher). Sterilized *A. thaliana* seeds were sown on the surface of agar-filled 5 mL pipette tips (Gilson) that had their tops trimmed to approximately 1 cm above the agar fill line and bottoms cut off after agar had solidified. Seeded and trimmed agar tips were seated into the open tops of glass vials containing soil-BH mixtures such that germinating plant roots grew downward through the agar plug and eventually into the soil medium below. Seeded agar tips were secured to glass vials with parafilm, placed into a humidified chamber, and grown on a growth rack (room temperature, 16 h/8 h day/night, 150 μmol·m^-2^·s^-1^ light intensity).

#### Study participant details

This study did not involve human participants.

### Method details

#### Prion uptake and translocation

##### Root uptake of fluorescently tagged PrP^res^ via confocal microscopy

*Arabidopsis thaliana* was grown by the vertical or horizontal plate agar method (*vide supra*) for 1-2 weeks. Seedling roots were then incubated for 24 h in 10^-1.3^ or 10^-2.3^ g·mL^-1^ RML-infected or non-infected BH (mouse), that had been processed by the prion enrichment protocol described above and fluorescently tagged using the Alexa Fluor® 488 carboxylic acid, succinimidyl ester fluorophore (Life Technologies) per manufacturer’s directions. Following incubation, plant roots were removed from solution, rinsed extensively with plant buffer solution, and imaged using a confocal microscope. Intact roots from 1- to 2-week-old seedlings were wet-mounted between two 35×50 mm (thickness 1) sheets of coverglass (Fisher) with plant buffer solution. A confocal laser scanning microscope (Nikon Instruments A1R-A1) was used with 10× and 20× dry and 60× and 100× oil-immersion objectives. Fluorophore-conjugated PrP^res^ was visualized by excitation with an argon laser at 488 nm and detected at 500-550 nm with a band-path filter.

##### Measurement of PrP^TSE^ translocation to aerial tissues via mb-PMCA

All plants used in these experiments were grown using the Ice-Cap or soil culture methods (*vide supra*) with or without PrP^TSE^ (HY). Duration of growth on prion-containing media (or non-infected media for controls) varied by plant species: 4 d for Ice-Cap-grown and 22 d for soil-grown *A. thaliana*, 25 d for Ice-Cap-grown alfalfa, 25 d for Ice-Cap-grown barley. Following these incubation periods, aerial tissues of plants were harvested by cutting them away from the top surface of the agar plugs in 96-well plates (or 5 mL pipette tips for soil-grown *A. thaliana*) using scissors or blades never before used for prion work. Stem and leaf tissues were stored at –80°C until analysis.

#### Intracerebral challenge mouse bioassay

##### Generation of plant material for intracerebral challenge

Only *A. thaliana* were used in these experiments and all were grown using the horizontal plate agar culture and 6-well plate liquid culture methods (*vide supra*). *Arabidopsis* plants grown on agar were harvested after four weeks of growth on horizontal plate culture. Hydroponically-grown *Arabidopsis* were germinated on vertical or horizontal agar plates for four weeks and then transferred to the 6-well plate liquid culture system and allowed to grow on prion-containing (RML) media (or non-infected media for controls) for 4 days. Following exposure incubation, aerial tissues were harvested by cutting them away from roots, above either the sterile agar surface (for horizontal plate agar culture) or the parafilm bridge (six-well plate liquid culture) using scissors or blades never before used for prion work. Stem and leaf tissues were stored at –80°C until assayed.

##### Preparation and administration of plant material via intracerebral inoculation

Following harvest, *A. thaliana* were separated into stem and leaf tissues and homogenized to 10^-0.77^ g·mL^-1^ and 10^-0.25^ g·mL^-1^, respectively, in PBS at pH 7.4 containing non-infected BH (mouse) at 10^-1.0^ g·mL^-1^. A 20 μL aliquot of plant homogenate was intracerebrally inoculated into anesthetized 4-week-old male CD-1 mice. Mice were monitored daily for disease progression and euthanized upon development of clinical signs consistent with a TSE (including kyphosis, lethargy, poor balance, and lack of motivation to acquire food and water). Brains from clinically diseased animals were harvested immediately post-mortem and divided sagittally with half prepared for evaluation by immunoblot. All challenged animals were tested for the presence of PrP^TSE^ in brain tissue by immunoblotting. Survival time was defined as the number of days post-inoculation to euthanization. Animals that succumbed to intercurrent illness within the first 10 days post-inoculation were excluded from data analysis.

#### Oral challenge mouse bioassay

##### Preparation and administration of plant material for exposure via oral gavage

*A. thaliana* plants grown by the Ice-Cap method (*vide supra*) with or without PrP^TSE^ (RML) were homogenized in PBS at 10^-0.27^ g·mL^-1^, and 200 μL were orally gavaged into 4- to 6-week-old male CD-1 mice as either a single dose (each mouse received the equivalent of ∼100 mg dry weight plant tissue) or the same dose each day for five consecutive days. In a separate experiment (Figure S7), designed to determine the effect of plant material on infectivity, a dilution series was also carried out in which 150 μL of RML-infected mouse BH in PBS at 10^-3.0^ to 10^-1.0^ g·mL^-1^ was administered with or without 100 mg (dry) non-infected *A. thaliana* homogenates made from combined stems and leaves. The plant co-administration experiment (Figure S7) was terminated at 455 days for practical reasons.

##### Preparation and administration of plant material for exposure via *ad libitum* feeding

Aerial tissues from alfalfa or barley grown by the Ice-Cap method (*vide supra*) in media containing PrP^TSE^ (RML) or non-infected brain homogenate were chopped into square pieces (13 mm or smaller), and 0.14 g plant tissue per mouse was free-fed to 4- to 6-week-old male CD-1 mice every day for 30 days.

Following oral challenges, mice were monitored daily for disease progression and were euthanized upon development of clinical signs consistent with a TSE. Brain and spleen tissue was harvested, stored, and analyzed via mb-PMCA (*vide supra*). Statistical differences in the prevalence of clinical disease or PrP^TSE^-positivity between mice fed plants grown in contaminated media and mice fed plants grown in non-infected media were determined via the one-sided Fisher’s exact test (Prism).

### Quantification and statistical analysis

#### Amplification of PrP^TSE^ by protein misfolding cyclic amplification assay (PMCA)

##### Generation of normal brain substrate for PMCA

Non-infected Golden Syrian hamsters or CD-1 mice were euthanized by carbon dioxide asphyxiation and immediately perfused with Dulbecco’s phosphate buffered saline (DPBS) without Ca^2+^ or Mg^2+^ (Thermo) modified with 5 mM EDTA. After perfusion, brains were immediately removed, flash frozen in liquid nitrogen, and stored at –80°C until use. Brains were homogenized on ice at 10^-1^ g·mL^-1^ using one of three methods: (1) pestle grinder with disposable attachment (Fisher), (2) Bullet Blender Storm 24 bead beater (Next Advance), or (3) Dounce homogenization (Kimble Chase) in ice-cold PMCA conversion buffer (DPBS supplemented with 150 mM NaCl, 1% Triton X-100, 5 mM EDTA, and EDTA-free protease inhibitor cocktail (Roche; 7 mini tablets or 1 regular tablet per 50 mL of conversion buffer). The homogenate was clarified by centrifugation for 2 minutes at 2,000 *g*. The supernatant was transferred to pre-chilled microcentrifuge tubes and stored at –80°C until use.

##### Microplate-based protein misfolding cyclic amplification (mb-PMCA)

The mb-PMCA assay was used to detect PrP^TSE^ for all experiments except the determination of plant matrix effects on PrP^TSE^ detection, for which PMCAb was used (*vide infra*). The mb-PMCA assay was used as previously described.^22^^,^^28^ Briefly, 4 μL test sample was added to 36 μL normal brain substrate with one Teflon® bead (McMaster-Carr) in a 96-well microplate (Axygen). Microplates were covered with a sealing mat (Axymat) and placed on a rack inside a Misonix S-4000 microplate horn and the reservoir was filled with 300 mL water. For analysis of hamster-derived PrP^TSE^, one round of mb-PMCA consisted of 96 cycles of 10 s sonication at 70% of maximum power followed by 29 minutes 50 seconds of incubation at 37°C. For analysis of mouse-derived PrP^TSE^, one round of mb-PMCA consisted of 96 cycles of 20 s sonication at 70% of maximum power followed by 29 minutes 40 seconds of incubation at 37°C. Subsequent rounds of mb-PMCA were generated by repeating this sonication with 4 μL of the mb-PMCA products from the previous round with 36 μL fresh normal brain substrate and a new Teflon® bead. Proteinase K-resistant reaction products were visualized by immunoblot (*vide infra*).

##### Protein misfolding cyclic amplification with beads (PMCAb)

The PMCAb assay^30^ was used to determine the matrix effect of plant homogenates on PrP^TSE^ amplification and detection. The PMCAb method uses a larger volume of test material than mb-PMCA (10 μL *vs* 4 μL), which facilitated the additional volume of the spiked plant homogenate in this experiment. Thin-walled PCR tubes containing 10 μL test sample, 90 μL normal brain substrate, and 2 Teflon® beads (McMaster-Carr) were placed in a Misonix S-4000 microplate horn and the reservoir was filled with 300 mL water. Each round of PMCAb was subjected to 96 cycles, each consisting of 10 s sonication at 80% of maximum power followed by 29 min 50 s incubation at 37°C. For serial PMCAb, at the conclusion of one 96-cycle round of PMCAb, 10 μL of each sample was transferred to 90 μL fresh normal brain substrate and subjected to another 96-cycle round of PMCAb. Proteinase K-resistant reaction products were visualized by immunoblot (*vide infra*).

##### Protein misfolding cyclic amplification (as performed by the laboratory of Rodrigo Morales)

Brains from wild-type mice were homogenized at 10^-1.0^ g·mL^-1^ in PMCA conversion buffer (PBS 1× supplemented with 150 mM NaCl, 1% Triton X-100 and a cocktail of protease inhibitors (Complete™ Protease Inhibitor Cocktail (with EDTA) Roche)), centrifuged (805 *g*, 1 min, 4°C), and stored at −80°C until use. Ninety μL PMCA substrate aliquots were mixed with 10 μL of brain or spleen homogenates and added to 0.2-mL PCR tubes containing three Teflon beads. These mixtures were subjected to 96 PMCA cycles (29 min 40 s incubation followed by 20 s sonication, 37°C). For subsequent rounds of 96 PMCA cycles, an aliquot of the amplified material was diluted 10-fold into fresh brain substrate. Products from the third PMCA round were digested with 100 mg·mL^-1^ proteinase K for 60 min at 37°C with shaking in an Eppendorf® thermomixer (450 rpm). The digestion reaction was stopped by adding lithium dodecyl sulfate buffer and heating to 95°C for 10 min. Samples were then fractionated via gel electrophoresis using 4-12% NuPAGE Bis-Tris precast gels (Invitrogen), transferred to a nitrocellulose membrane, and analyzed via western blot. Membranes were incubated overnight at 4°C using the 6D11 anti-PrP antibody at a 1:30,000 dilution (BioLegend). For assay controls, each PMCA reaction set included serial dilutions of an RML-infected brain homogenate of known seeding activity and four unseeded PMCA tubes were included as negative controls.

#### Detection of PrP^res^ by immunoblotting

For mb-PMCA, PMCAb, and bioassay samples, 20 μL of each PMCA reaction or 20 μL of 10^-1.0^ g·mL^-1^ BH from brains of clinically diseased rodents in PBS, was transferred into a thin-walled PCR tube and treated with 100 μg·mL^-1^ PK (Mo Bio Laboratories) and 0.04 g·mL^-1^ SDS in PBS and incubated for 1 h at 37°C with shaking (1000 rpm). Following PK digestion, samples were treated with additions of 4× LDS sample buffer and 10× NuPAGE reducing agent (Life Technologies, Grand Island, NY), each to final concentrations of 1×, and were then heated for 5 min at 95°C. A 30 μL aliquot of each sample was resolved on 12% NuPAGE Bis-Tris gels (Life Technologies) for 50 min at 200 V and then transferred to a polyvinyl difluoride (PVDF) membrane for 60 min at 25 V. The PVDF membranes were blocked for 1 h in 5% powdered milk in Tris-buffered saline plus Tween-20 (TBST), followed by application of the primary antibody SAF-83 for mouse samples (Cayman Chemical Company) and 3F4 for hamster samples (Millipore) diluted 1:10,000 in 5% powdered milk in TBST, for 1 h. The PVDF membranes were then rinsed 3× with TBST and incubated for 1 h with the secondary sheep anti-mouse immunoglobulin G conjugated with horseradish peroxidase (1:10,000 dilution in 5% milk-TBST). Immunoreactivity was detected using an enhanced chemiluminescent detection system (Thermo Scientific) in an EC3 imaging system (UVP Bioimaging Systems). Densitometry was performed using VisionWorks LS software version 6.6a (UVP Bioimaging Systems). Samples displaying ≥5 × densitometric signal of the background were considered positive, as previously described.^45^

#### Effect of plant homogenate on the limits of PrP^TSE^ detection

We employed the PMCAb assay (*vide supra*) to test the effect of plant tissue homogenates on PrP^TSE^ detection in dilutions of HY-infected hamster brain homogenate (g·mL^-1^). Plant tissue homogenates were prepared from aerial tissues harvested from plants grown via the Ice-Cap method or on soil. Stem and/or leaf tissues were homogenized to 10^−-0.5^ to 10^-0.3^ g·mL^-1^ in conversion buffer by flash freezing tissues with liquid nitrogen, grinding with mortar and pestle, and subsequently homogenizing using the bead beater (Next Advance). Normal brain substrate (90 μL) was seeded with 8 μL of the desired dilution of infected BH in the absence (+ 2 μL PMCA buffer) or presence (+ 2 μL 10^-0.3^ g·mL^-1^ plant homogenate) of plant tissue in 0.2 mL thin-walled PCR tubes with two 2.38 mm Teflon® beads (McMaster-Carr). The PMCAb assay was carried out for seven rounds.

#### Statistical analysis

Details of statistical analyses can be found in their respective tables and figures. For infection rates of mice orally challenged with aerial tissues of *A. thaliana*, alfalfa, or barley grown in media containing sequentially degraded PrP^TSE^-infected or normal brain tissue, see Table 1. For the fraction of plants with detectable PrP^TSE^ in aerial tissues (via mb-PMCA) after growth in media containing sequentially degraded PrP^TSE^-infected or normal brain homogenate (BH), see Table S1. For the characterization of the sequentially degraded material infectivity via hamster intracerebral inoculation, see Figure S2. GraphPad Prism was used for all statistical analyses.

## References

[1] Colby D.W., Prusiner S.B. (2011). Prions. Cold Spring Harbor Perspect. Biol..

[2] Schneider K., Fangerau H., Michaelsen B., Raab W.H.M. (2008). The early history of the transmissible spongiform encephalopathies exemplified by scrapie. Brain Res. Bull..

[3] Cassmann E.D., Greenlee J.J. (2020). Pathogenesis, detection, and control of scrapie in sheep. Am. J. Vet. Res..

[4] Collinge J. (1999). Variant Creutzfeldt-Jakob disease. Lancet.

[5] Williams E.S., Young S. (1980). Chronic wasting disease of captive mule deer: a spongiform encephalopathy. J. Wildl. Dis..

[6] Kim T.Y., Shon H.J., Joo Y.S., Mun U.K., Kang K.S., Lee Y.S. (2005). Additional cases of chronic wasting disease in imported deer in Korea. J. Vet. Med. Sci..

[7] Sohn H.J., Kim J.H., Choi K.S., Nah J.J., Joo Y.S., Jean Y.H., Ahn S.W., Kim O.K., Kim D.Y., Balachandran A. (2002). A case of chronic wasting disease in an elk imported to Korea from Canada. J. Vet. Med. Sci..

[8] Benestad S.L., Mitchell G., Simmons M., Ytrehus B., Vikøren T. (2016). First case of chronic wasting disease in Europe in a Norwegian free-ranging reindeer. Vet. Res..

[9] Miller M.W., Williams E.S. (2003). Prion disease: horizontal prion transmission in mule deer. Nature.

[10] Mathiason C.K., Powers J.G., Dahmes S.J., Osborn D.A., Miller K.V., Warren R.J., Mason G.L., Hays S.A., Hayes-Klug J., Seelig D.M. (2006). Infectious prions in the saliva and blood of deer with chronic wasting disease. Science.

[11] Mathiason C.K., Hays S.A., Powers J., Hayes-Klug J., Langenberg J., Dahmes S.J., Osborn D.A., Miller K.V., Warren R.J., Mason G.L., Hoover E.A. (2009). Infectious prions in pre-clinical deer and transmission of chronic wasting disease solely by environmental exposure. PLoS One.

[12] Miller M.W., Williams E.S., Hobbs N.T., Wolfe L.L. (2004). Environmental sources of prion transmission in mule deer. Emerg. Infect. Dis..

[13] Pedersen J., Somerville R.A., Riesner D., Deslys J.P., Pocchiari M., Somerville R.A. (2012). Decontamination of Prions.

[14] Taylor D.M. (1999). Inactivation of prions by physical and chemical means. J. Hosp. Infect..

[15] Taylor D.M. (2000). Inactivation of transmissible degenerative encephalopathy agents: a review. Vet. J..

[16] Taylor D.M., Diprose M.F. (1996). The response of the 22A strain of scrapie agent to microwave irradiation compared with boiling. Neuropathol. Appl. Neurobiol..

[17] Brown P., Rau E.H., Lemieux P., Johnson B.K., Bacote A.E., Gajdusek D.C. (2004). Infectivity studies of both ash and air emissions from simulated incineration of scrapie-contaminated tissues. Environ. Sci. Technol..

[18] Seidel B., Thomzig A., Buschmann A., Groschup M.H., Peters R., Beekes M., Terytze K. (2007). Scrapie Agent (Strain 263K) can transmit disease via the oral route after persistence in soil over years. PLoS One.

[19] Brown P., Gajdusek D.C. (1991). Survival of scrapie virus after 3 years' interment. Lancet.

[20] Georgsson G., Sigurdarson S., Brown P. (2006). Infectious agent of sheep scrapie may persist in the environment for at least 16 years. J. Gen. Virol..

[21] Johnson C.J., Pedersen J.A., Chappell R.J., McKenzie D., Aiken J.M. (2007). Oral transmissibility of prion disease is enhanced by binding to soil particles. PLoS Pathog..

[22] Plummer I.H., Johnson C.J., Chesney A.R., Pedersen J.A., Samuel M.D. (2018). Mineral licks as environmental reservoirs of chronic wasting disease prions. PLoS One.

[23] Icoz I., Andow D., Zwahlen C., Stotzky G. (2009). Is the Cry1Ab protein from Bacillus thuringiensis (Bt) taken up by plants from soils previously planted with Bt corn and by carrot from hydroponic culture?. Bull. Environ. Contam. Toxicol..

[24] Paungfoo-Lonhienne C., Lonhienne T.G.A., Rentsch D., Robinson N., Christie M., Webb R.I., Gamage H.K., Carroll B.J., Schenk P.M., Schmidt S. (2008). Plants can use protein as a nitrogen source without assistance from other organisms. P Natl Acad Sci USA.

[25] Rasmussen J., Gilroyed B.H., Reuter T., Dudas S., Neumann N.F., Balachandran A., Kav N.N.V., Graham C., Czub S., McAllister T.A. (2014). Can plants serve as a vector for prions causing chronic wasting disease?. Prion.

[26] Pritzkow S., Morales R., Moda F., Khan U., Telling G.C., Hoover E., Soto C. (2015). Grass plants bind, retain, uptake, and transport infectious prions. Cell Rep..

[27] Shikiya R.A., Bartz J.C. (2011). In vitro generation of high-titer prions. J. Virol..

[28] Moudjou M., Sibille P., Fichet G., Reine F., Chapuis J., Herzog L., Jaumain E., Laferrière F., Richard C.A., Laude H. (2013). Highly infectious prions generated by a single round of microplate-based protein misfolding cyclic amplification. mBio.

[29] Bessen R.A., Marsh R.F. (1992). Identification of two biologically distinct strains of transmissible mink encephalopathy in hamsters. J. Gen. Virol..

[30] Johnson C.J., Aiken J.M., McKenzie D., Samuel M.D., Pedersen J.A. (2012). Highly efficient amplification of chronic wasting disease agent by protein misfolding cyclic amplification with beads (PMCAb). PLoS One.

[31] Castilla J., Saá P., Morales R., Abid K., Maundrell K., Soto C. (2006). Amyloid, Prions, and Other Protein Aggregates.

[32] Clark K.A., Krysan P.J. (2007). Protocol: An improved high-throughput method for generating tissue samples in 96-well format for plant genotyping (Ice-Cap 2.0). Plant Methods.

[33] Johnson C.J., Phillips K.E., Schramm P.T., McKenzie D., Aiken J.M., Pedersen J.A. (2006). Prions adhere to soil minerals and remain infectious. PLoS Pathog..

[34] Chandler R.L. (1961). Encephalopathy in mice produced by inoculation with scrapie brain material. Lancet.

[35] Castro-Camus E., Palomar M., Covarrubias A.A. (2013). Leaf water dynamics of Arabidopsis thaliana monitored in-vivo using terahertz time-domain spectroscopy. Sci. Rep..

[36] Denkers N.D., Hoover C.E., Davenport K.A., Henderson D.M., McNulty E.E., Nalls A.V., Mathiason C.K., Hoover E.A. (2020). Very low oral exposure to prions of brain or saliva origin can transmit chronic wasting disease. PLoS One.

[37] Diringer H., Roehmel J., Beekes M. (1998). Effect of repeated oral infection of hamsters with scrapie. J. Gen. Virol..

[38] Bolton D.C., Rudelli R.D., Currie J.R., Bendheim P.E. (1991). Copurification of Sp33-37 and scrapie agent from hamster brain prior to detectable histopathology and clinical disease. J. Gen. Virol..

[39] Raymond G.J., Chabry J., Lehmann S., Grassi J. (2004). Techniques in Prion Research.

[40] Johnson C.J., Bennett J.P., Biro S.M., Duque-Velasquez J.C., Rodriguez C.M., Bessen R.A., Rocke T.E. (2011). Degradation of the disease-associated prion protein by a serine protease from lichens. PLoS One.

[41] Murashige T., Skoog F. (1962). A revised medium for rapid growth and bio assays with tobacco tissue cultures. Physiol. Plantarum.

[42] Linsmaier E.M., Skoog F. (1965). Organic growth factor requirements of tobacco tissue cultures. Physiol. Plantarum.

[43] Krysan P. (2004). Ice-cap. A high-throughput method for capturing plant tissue samples for genotype analysis. Plant Physiol..

[44] Su S.H., Clark K.A., Gibbs N.M., Bush S.M., Krysan P.J. (2011). Ice-Cap: a method for growing Arabidopsis and tomato plants in 96-well plates for high-throughput genotyping. J. Vis. Exp..

[45] Chen B., Morales R., Barria M.A., Soto C. (2010). Estimating prion concentration in fluids and tissues by quantitative PMCA. Nat. Methods.

