# Supplementary material for: Plants as vectors for environmental prion transmission
Source: iScience. 2023 Nov 10;26(12):108428. doi: 10.1016/j.isci.2023.108428 (PMC10700824; doi:10.1016/j.isci.2023.108428)
Supplement: Document S1. Figures S1–S7 and Table S1 [file mmc1.pdf]

iScience, Volume *26*

Supplemental information

Plants as vectors for environmental prion transmission

Christina M. Carlson, Samuel Thomas, Matthew W. Keating, Paulina Soto, Nicole M. Gibbs, Haeyoon Chang, Jamie K. Wiepz, Annabel G. Austin, Jay R. Schneider, Rodrigo Morales, Christopher J. Johnson, and Joel A. Pedersen

Supplemental Figures and Tables

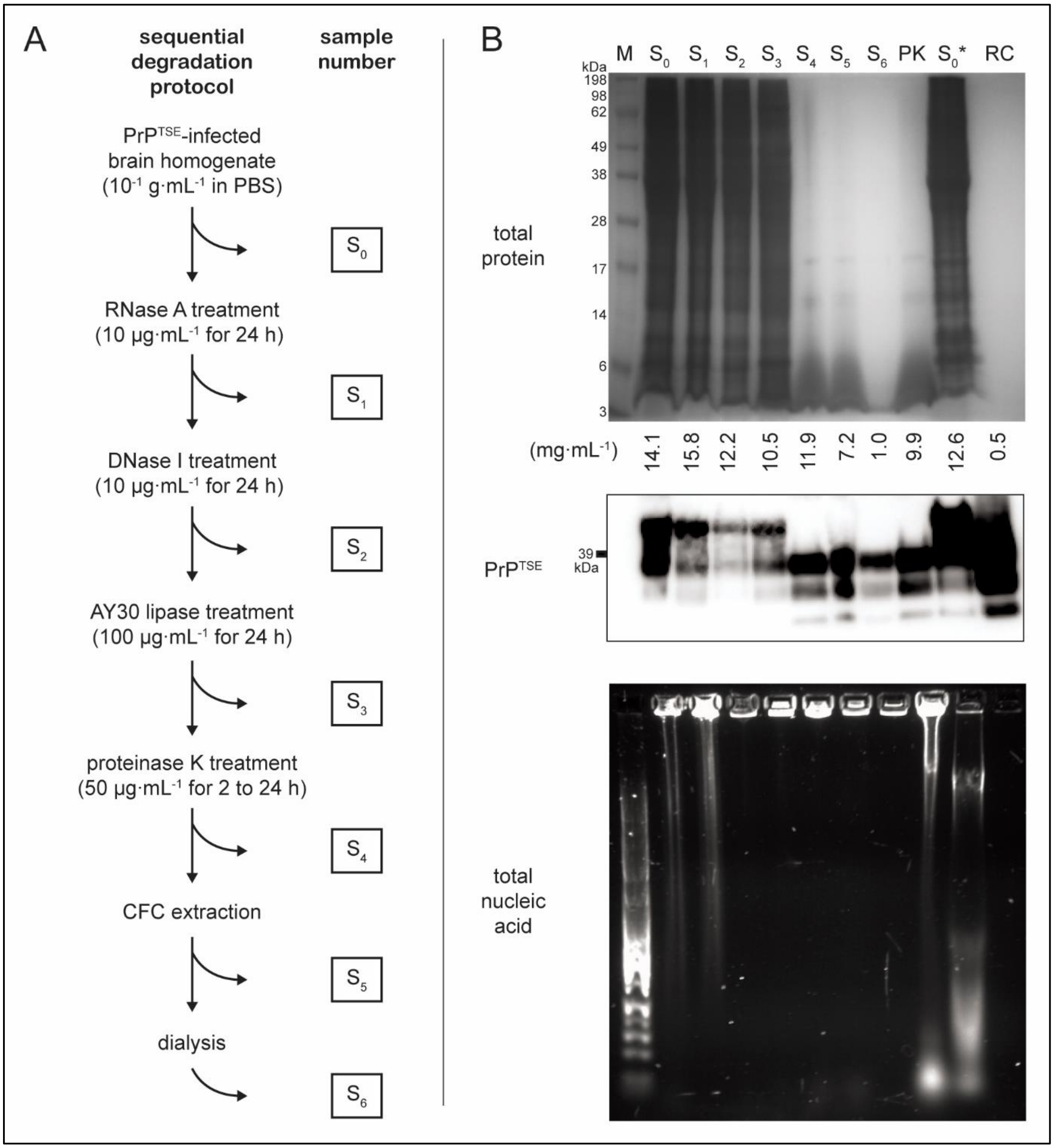

Figure S1. The sequential degradation protocol markedly depletes proteinase K-labile proteins and total nucleic acids, while retaining strong PrP^{TSE} immunoreactivity; related to STAR Methods. (**A**) Schematic of the degradation protocol designed to more closely mimic environmental decomposition than typical proteinase K digestion (PK) or Raymond and Chabry preparation (RC). (**B**) Total proteins are depleted following proteinase K digestion (top panel; SDS-PAGE with Coomassie stain; quantified via bicinchoninic assay), while PrP^{TSE} is well-retained (center panel; anti-PrP antibody 3F4), and total nucleic acids are depleted following nuclease treatment (bottom panel; agarose electrophoresis with SYBR Green staining). Results were reproduced in at least three independent experiments, each using different hamster brains. Abbreviations: CFC, chlorofluorocarbon; PK, proteinase K; M, molecular weight marker; RC:

Raymond and Chabry preparation (see methods); S_n, sample number; S_0*, replicate brain homogenate from additional animal.

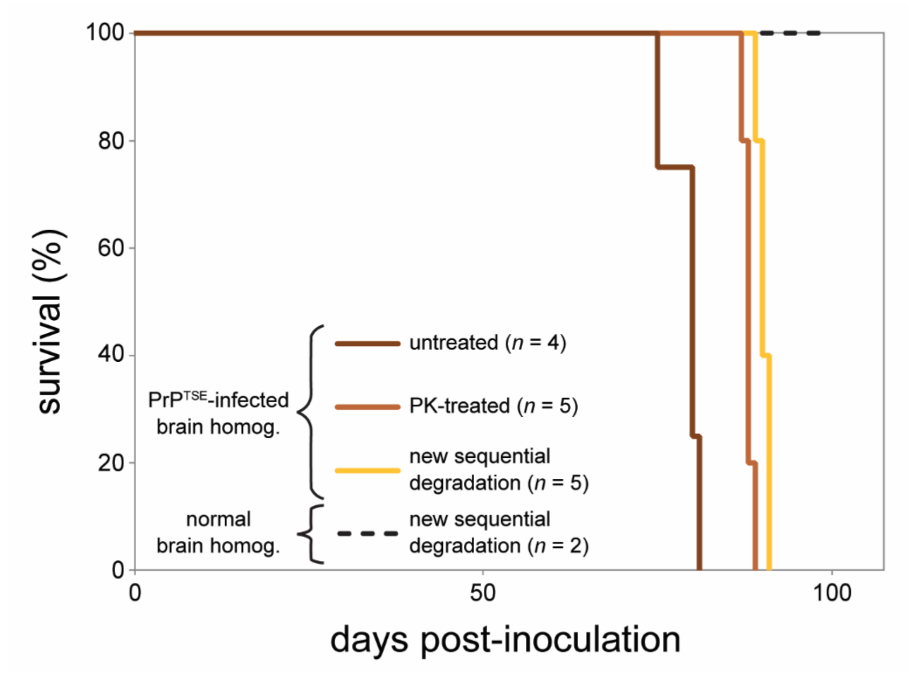

Figure S2. The sequential degradation procedure results in an enrichment retaining nearly the same infectivity as the typical proteinase K digestion; related to STAR Methods. Survival curves of hamsters intracerebrally inoculated with 10^{-1} g·mL^{-1} PrP^{TSE}-infected brain homogenates prepared three ways: untreated (median 80 d; *n* = 4), digested with proteinase K (PK; median 88 d; *n* = 5), and sequentially degraded via the new method (median 90 d; *n* = 5); survival analysis via Mantel-Cox log rank test *p*-value < 0.001 for comparison of all curves (3 degrees of freedom).

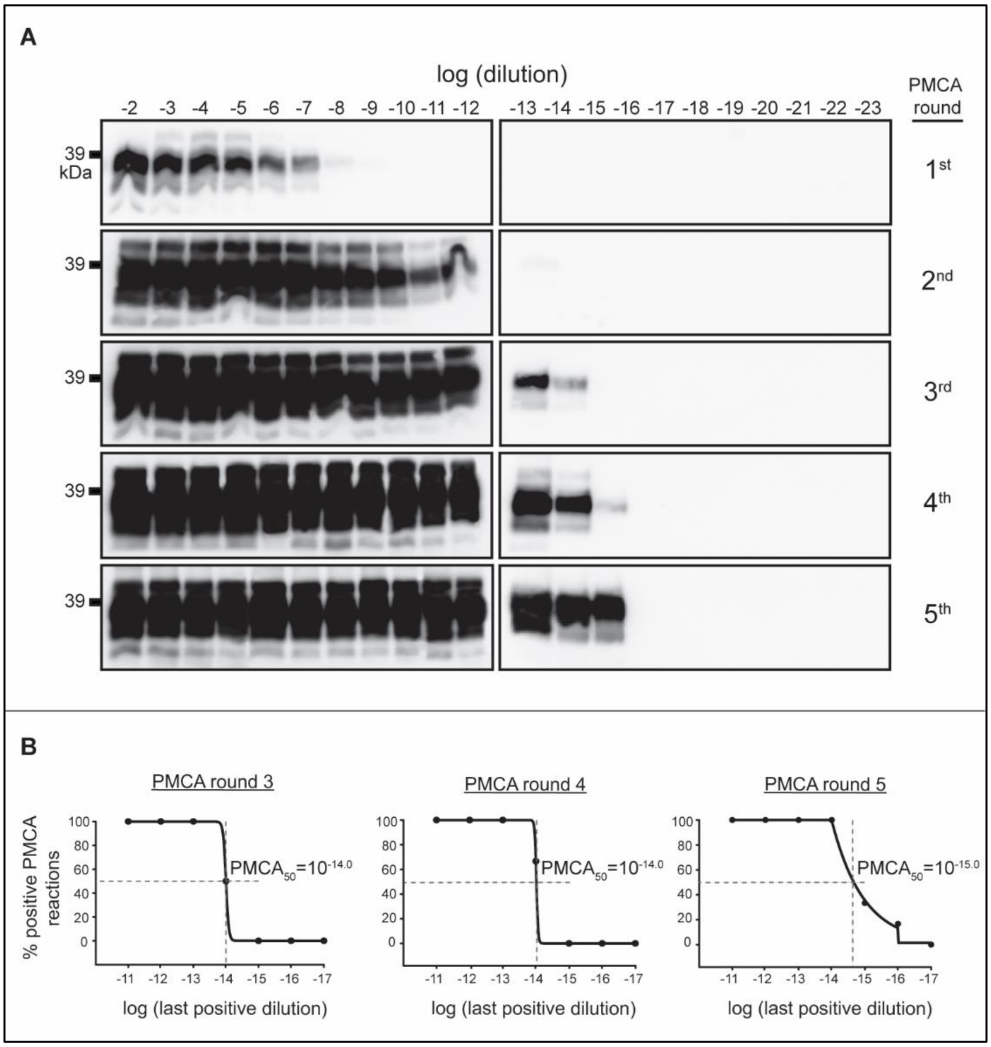

Figure S3. Determination of lower limit of detection for PrP^{TSE} by serial microplate-based protein misfolding cyclic amplification (mb-PMCA) assay; related to STAR Methods. (**A**) Representative immunoblot series from six independent experiments illustrating the endpoint for detection of HY PrP^{res} after five 96-cycle serial mb-PMCA rounds lies near 10^{-15} dilutions of HY brain homogenate ($g{\cdot}mL^{-1}$; prepared in 1× DPBS). (**B**) Serial mb-$PMCA_{50}$ values for rounds 3, 4, and 5. Abbreviations: DPBS, Dulbecco's phosphate buffered saline, HYBH, brain homogenate from end-stage hamsters infected with the Hyper prion strain; mb-PMCA, microplate-based protein misfolding cyclic amplification; $PMCA_{50}$, dilution at which half of the replicates produced positive signal for PrP^{res}; PrP^{res}, proteinase K-resistant prion protein.

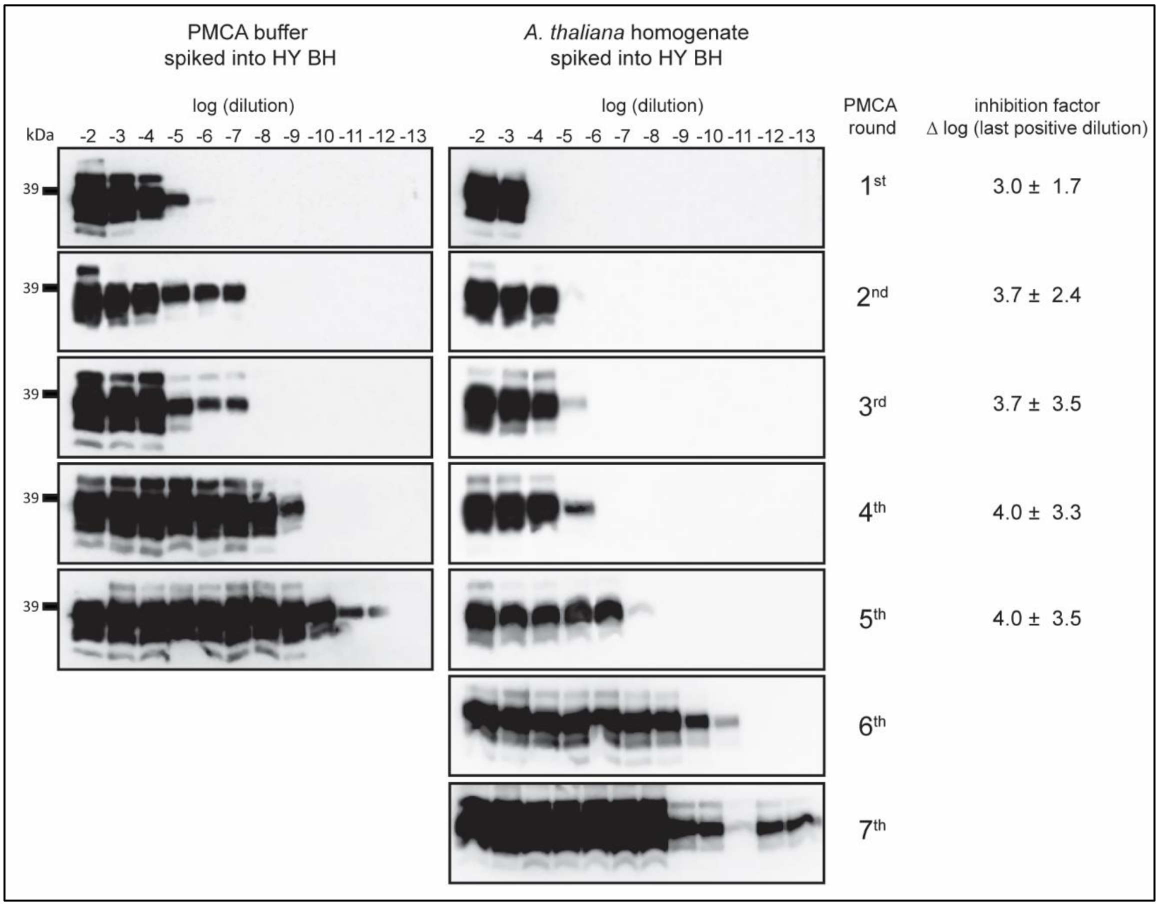

Figure S4. Effect of *Arabidopsis thaliana* homogenate on PrP^{TSE} detection by PMCAb; related to STAR Methods. Representative immunoblots showing serial amplification of hamster HY prions in dilutions of brain homogenate (HY BH; g·mL^{-1}) by PMCAb in the absence and presence of 10^{-1} g·mL^{-1} *A. thaliana* stem and leaf homogenate. Inhibition factor is the difference in lowest detected dilution in rounds 1 through 5, derived from an average of three replicate experiments.

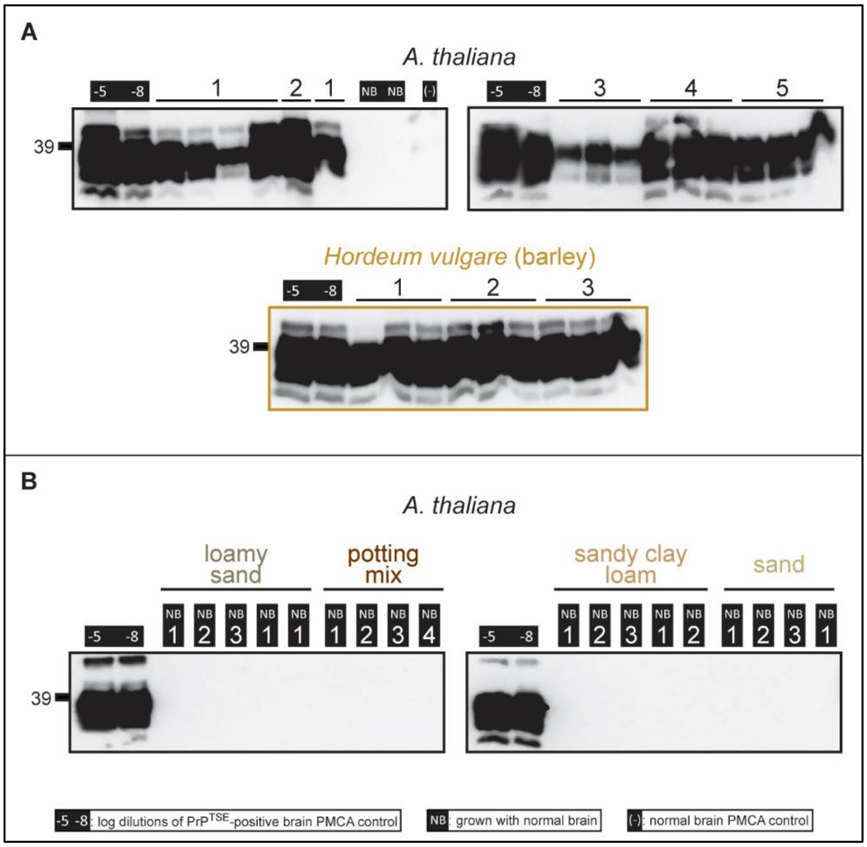

Figure S5. Additional evidence of plant uptake and translocation of PrP^{TSE}; related to Figure 2. (**A**) Aerial tissues of *Arabidopsis thaliana* and *Hordeum vulgare* (barley) grown hydroponically for 25 days in PrP^{TSE}-contaminated media contain detectable PrP^{TSE} (HY) via serial mb-PMCA (5th round shown). (**B**) No detection of PrP^{TSE} via serial mb-PMCA (5th round shown) for *Arabidopsis thaliana* aerial tissues grown for 22 days in sand, potting mix, loamy sand, or sandy clay loam amended with normal brain homogenate. Numbers above lanes indicate individual plants. Brain homogenates were added at $10^{-3.0}$ to $10^{-1.7}$ $g{\cdot}mL^{-1}$ liquid media and $10^{-1.7}$ $g{\cdot}mL^{-1}$ soil.

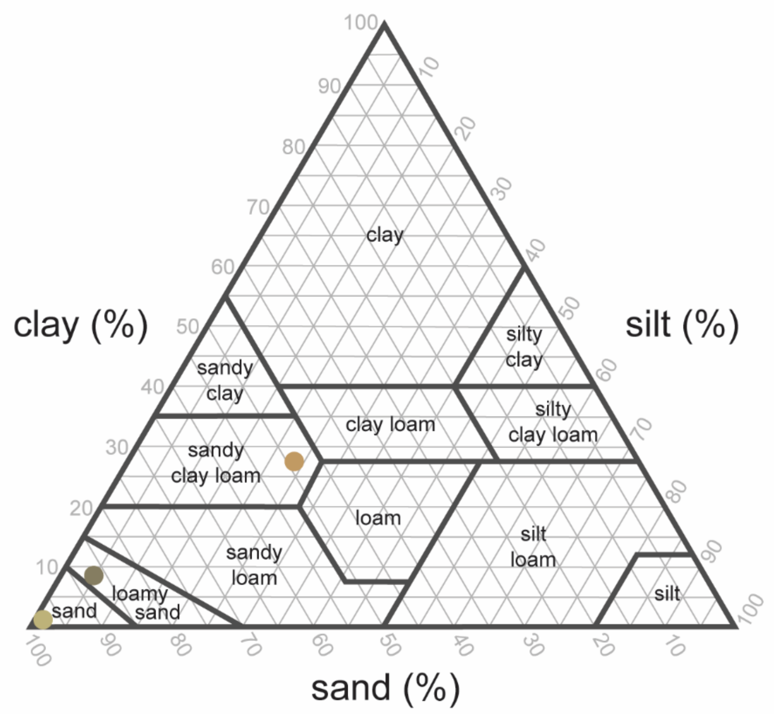

Figure S6. Soil texture triangle; related to Figure 2 and STAR Methods. Proportion of clay, silt, and sand of soils used for prion uptake experiments in *Arabidopsis thaliana*. Adapted from Natural Resources Conservation Service web-based soil texture calculator (accessed 08 November 2019): https://www.nrcs.usda.gov/wps/portal/nrcs/detail/soils/survey/?cid=nrcs142p2_054167

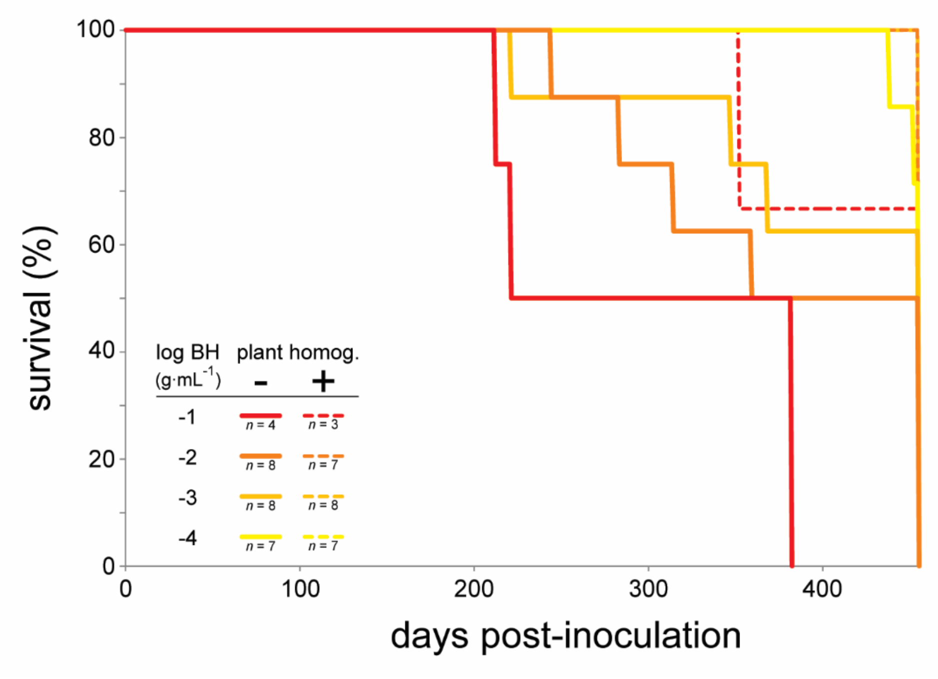

Fig. S7. Oral co-administration of untreated *A. thaliana* aerial tissues with PrP^{TSE}-infected brain homogenate (BH) decreases disease penetrance and lengthens incubation time; related to Figure 4 and Table 1. CD-1 mice were orally gavaged with dilutions of PrP^{TSE}-infected BH (10^{-1} to 10^{-4} g·mL^{-1}) with (dashed lines) or without (solid lines) 100 mg dry stem and leaf homogenate from axenically grown *A. thaliana* plants. Data are presented as survival curves; sample sizes indicated in figure legend. Curves representing groups with 100% survival across the course of the experiment overlay each other at the conclusion of the experiment (day 455).

Table S1. Fraction of plants with detectable PrP^{TSE} in aerial tissues (via mb-PMCA) after growth in media containing sequentially degraded PrP^{TSE}-infected or normal brain homogenate (BH); related to Figure 2.

Species	Media	PrP^{TSE}-positive plants (n/n_0)		*p*-value[a]
		PrP^{TSE}-infected BH	normal BH	
A. thaliana	hydroponic	9/9	0/33	< 0.001
	soil	8/12	0/13	< 0.001
alfalfa	hydroponic	1/2	0/9	< 0.001
barley	hydroponic	9/9	0/5	

[a] One-sided Fisher's exact test; crop species pooled
